# Amyloidosis-Related Cardiomyopathy Revealing Waldenstrom Macroglobulinemia: A Case Report

**DOI:** 10.7759/cureus.83278

**Published:** 2025-04-30

**Authors:** Manahil Tariq, Klara Lunte, Carole Fleury, Damien Logeart, Edouard Ballout, Virginie Siguret, Georges Jourdi

**Affiliations:** 1 Hematopathology, Lariboisière, AP-HP.Nord (Assistance Publique-Hôpitaux de Paris), Paris, FRA; 2 Hematopathology, Avicenne, AP-HP.Nord (Assistance Publique-Hôpitaux de Paris), Paris, FRA; 3 Cardiology, Lariboisière, AP-HP.Nord (Assistance Publique-Hôpitaux de Paris), Paris, FRA; 4 Hematopathology, Lariboisière, AP-HP.Nord (Assistance Publique-Hôpitaux de Paris) Université Paris Cité, Inserm U1144, Paris, FRA

**Keywords:** al amyloidosis, cardiac amyloidosis, infiltrative disease, lymphoplasmacytic lymphoma, waldenstrom macroglobulinemia

## Abstract

Cardiac amyloidosis is a rare infiltrative disease caused by the deposition of extracellular insoluble aggregates of misfolded proteins. Most cardiac amyloidosis seen in the clinic is due to either amyloid light-chain (AL) of immunoglobulins or transthyretin accumulation in various cardiac structures. AL amyloidosis is most often secondary to a monoclonal gammopathy of undetermined significance (MGUS) or multiple myeloma. More rarely, it is associated with lymphoplasmacytic lymphoma, a low-grade, mature, B-cell non-Hodgkin lymphoma. Here, we present a rare case of cardiac AL amyloidosis caused by Waldenstrom macroglobulinemia in a 70-year-old man. The clinical and biological characteristics of the patient, as well as the mutational profile, are detailed and discussed. This case report sheds light on the rare association of AL amyloidosis and Waldenstrom macroglobulinemia. It underlines the importance of the multidisciplinary collaboration between cardiologists, hematologists, clinical pathologists, and amyloidosis specialists to overcome diagnostic challenges using specific key tools encompassing immunofixation electrophoresis, mass spectrometry, immunophenotyping, cardiac magnetic resonance imaging, and next-generation sequencing. All of the above lead to the establishment of an early and appropriate diagnosis of such a rare clinical association, thus optimizing patient management.

## Introduction

Amyloidosis is a broad term that encompasses a group of diseases caused by deposits of misfolding proteins that consist of elongated, unbranched β-structures of separate monomers positioned perpendicular to the fibril axis and stacked strictly above each other, resulting in amyloid aggregates. Although over 40 proteins are known to be amyloidogenic to date, only 2 are known to have a tropism for cardiac tissues, namely, free immunoglobulin (Ig) light chains (AL) and transthyretin (ATTR). Cardiac AL amyloidosis is the most common subtype of systemic amyloidosis and has the worst prognosis compared to ATTR amyloidosis [1]. Abnormal Ig light chain folding results from either a proteolytic event or an amino acid sequence that renders the protein thermodynamically and kinetically unstable, leading to self-aggregation. AL amyloidosis leads to progressive organ dysfunction and organ failure and may eventually cause death. Approximately half of patients with AL amyloidosis already have heart disease at the time of diagnosis [2]. A high index of clinical suspicion is required for the diagnosis. Yet, the clinical symptoms are protean and deceitful [3]. The source of these toxic light chains can be a plasma cell clone or, less frequently (<10% of the cases), a lymphoplasmacytic lymphoma. Lymphoplasmacytic lymphoma is a rare type of low-grade mature B-cell non-Hodgkin lymphoma characterized by an admixture of small B lymphocytes, lymphoplasmacytoid cells, and plasma cells in the bone marrow. Its association with the IgM paraprotein is known as Waldenstrom macroglobulinemia (WM). Notable advancements in WM diagnosis and a better comprehension of the biology of WM have led to better clinical management. Here, we report a rare case of cardiac amyloidosis revealing a WM in order to provide clinicians and clinical pathologists further knowledge about the symptoms, imaging, and laboratory characteristics that should raise suspicion for this rare combination.

## Case presentation

**Figure 1:**
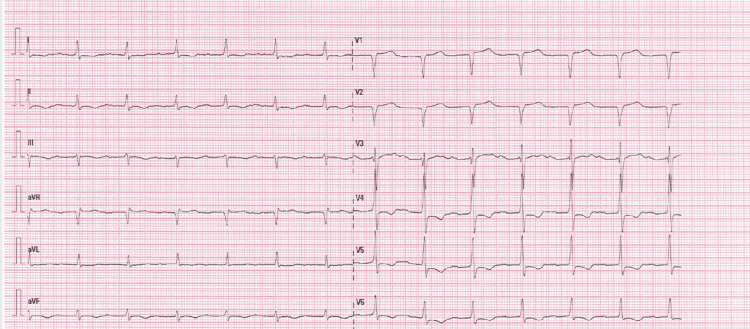
Electrocardiogram on hospital admission The electrocardiogram shows a type 1 atrioventricular block.

A 70-year-old man had a history of type 2 diabetes, arterial hypertension, and paroxysmal atrial fibrillation. His pre-admission long-term medications included metformin, gliclazide, dapagliflozin, furosemide, atorvastatin, apixaban, and amiodarone. He was admitted to the emergency room for exertional dyspnoea and a progressive onset of disabling lower limb edema. This clinical picture of congestive heart failure led to his hospitalization in the cardiology department. The electrocardiogram on admission showed a new-onset type 1 atrioventricular block (Figure 1).

**Figure 2:**
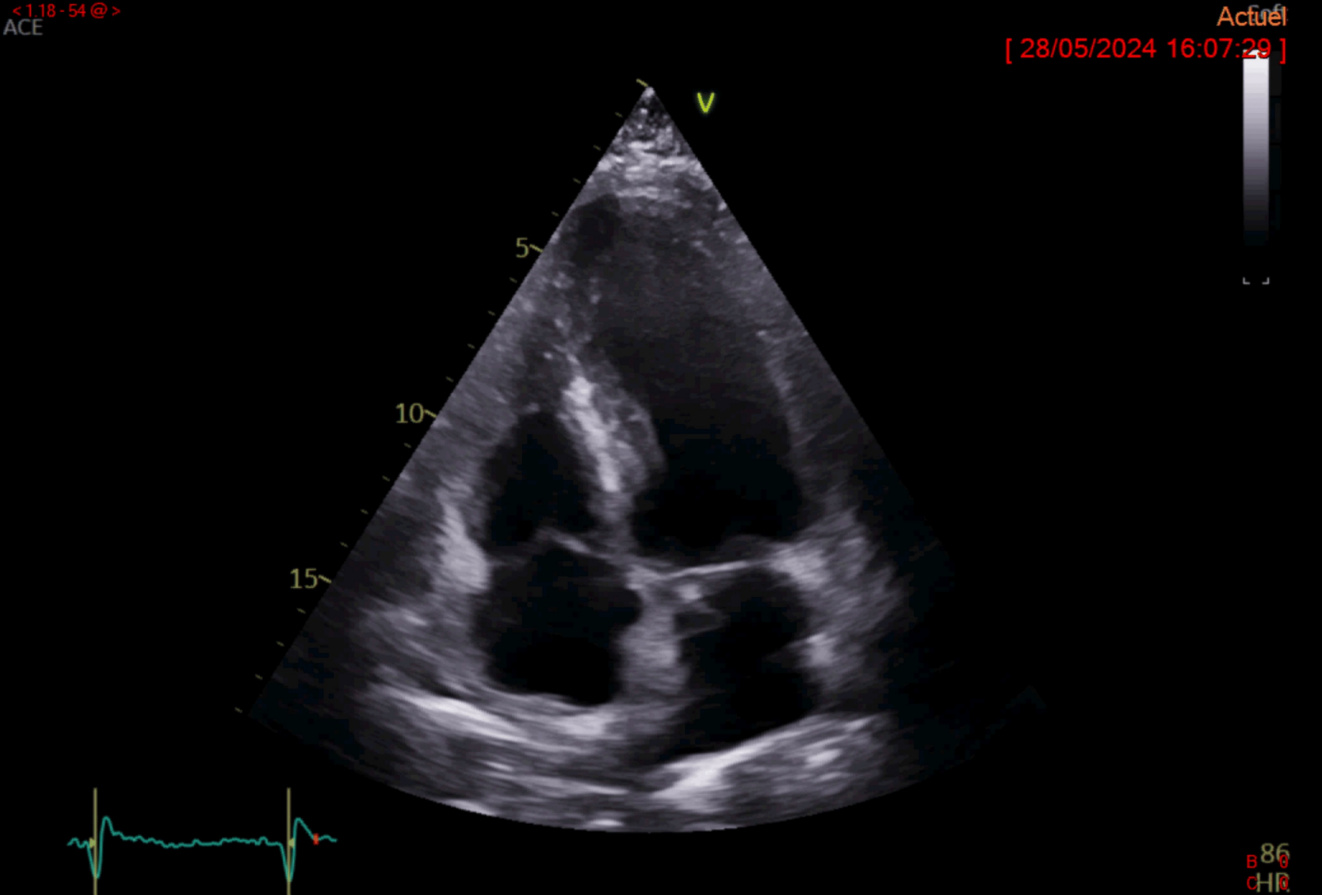
Transthoracic echocardiography Transthoracic echocardiography revealed concentric left ventricular hypertrophy with a septal diastolic thickness of 11.5 mm and a septal bulge thickness of 17 mm. The left ventricular ejection fraction (65%) was preserved.

Transthoracic echocardiography revealed concentric left ventricular hypertrophy (septal diastolic thickness of 11.5 mm and a septal bulge thickness of 17 mm) with a preserved left ventricular ejection fraction of 65% (Figure 2).

Laboratory tests performed upon admission revealed an increased N-terminal pro-B-type natriuretic peptide (NT-proBNP) at 1594 ng/L (Table 1). A thorough clinical examination revealed multiple organ damage with macroglossia and a numbness and tingling of the palmar aspect of the first three fingers of the left hand, suggestive of carpal tunnel syndrome, yet no nerve conduction study was performed.

**Table 1:** Blood test results on admission: biochemical, cytological, and hemostatic parameters An increased NT-proBNP level was noticed along with a slight decreased eGFR revealing an early-stage kidney disease of the patient. No other biological abnormality was recorded. NT-proBNP: N-terminal pro-B-type natriuretic peptide; eGFR: estimated glomerular filtration rate; CKD-EPI: Chronic Kidney Disease Epidemiology Collaboration

Parameters	Results	Normal Range
Sodium (mmol/L)	140	136-145
Potassium (mmol/L)	4.0	3.5-5.1
Chloride (mmol/L)	106	98-107
Bicarbonate (mmol/L)	23	23-31
Proteins (g/L)	67	64-83
Creatinine (µmol/L)	86	64-104
eGFR calculated by CKD-EPI * (mL/min/1.73 m^2^)	78.1	90-140
Troponin (ng/L)	29.2	<34.0
NT-proBNP * (ng/L)	1594	<300
White cell count (G/L)	7.2	4-10
Hemoglobin (g/dL)	12.6	13-17
Mean corpuscular volume (fL)	88.5	83-98
PNN (G/L)	5.13	1.5-7.0
Lymphocytes (G/L)	1.29	1.0-4.0
Monocytes (G/L)	0.61	0.2-1.0
Blood smear	No morphologically abnormal blood cells	Non-applicable
Platelet count (G/L)	172	150-450
Prothrombin time (PT, %)	81	70-120
Activated partial thromboplastin time (aPTT)	1.02	0.80-1.20
Factor II (U/dL)	72	70-130
Factor V (U/dL)	90	70-130
Factor X (U/dL)	73	70-130
Fibrinogen (g/L)	3.87	2.00-4.00

**Figure 3:**
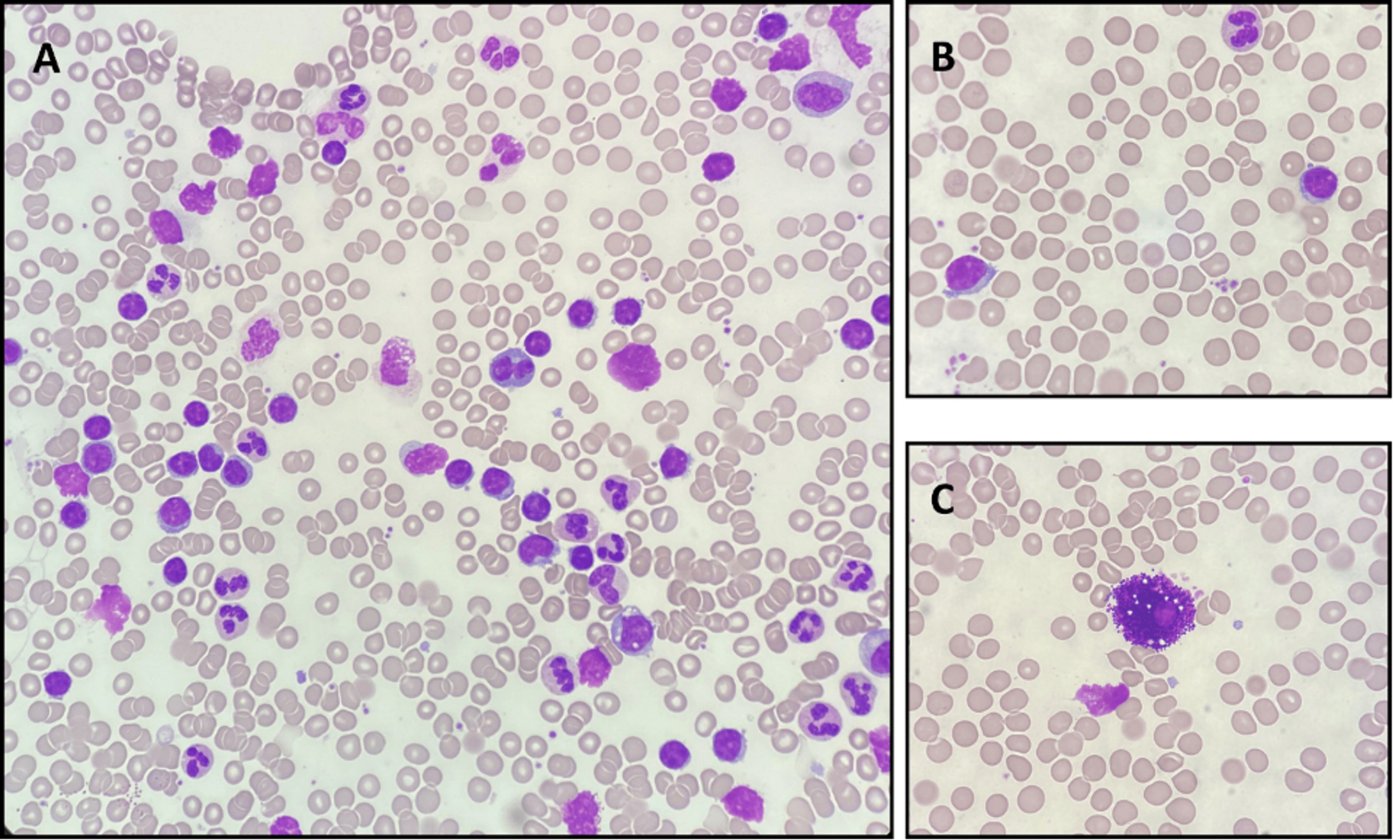
Bone marrow smear: May-Grünwald-Giemsa staining, x50 objective (A) and x100 (B and C) A lymphoid infiltration with 86% pleiomorphic lymphocytes, including small mature lymphocytes, nucleolated lymphocytes, and lymphoplasmocytes with basophilic cytoplasm and eccentric nucleus, was reported (3A). Some lymphocytes had small cytoplasmic buds and/or some fine villi, which is characteristic of Waldenstrom macroglobulinemia (3B). Many mast cells (hypogranular and vacuolated) were also observed (3C).

Serum protein electrophoresis revealed a homogeneous spike-like peak, quantified at 4.40 g/L, in the focal region of the gamma-globulin zone. Serum kappa and lambda free light chain levels were estimated at 40 mg/L (normal range: 3.3-19.4) and 332 mg/L (normal range: 5.7-26.3), respectively, with a kappa/lambda ratio of 0.12 (normal range: 0.26-1.65). Serum immunoassay confirmed the presence of a lambda IgM peak, thus establishing the diagnosis of a monoclonal gammopathy, while urinary immunoassay revealed the presence of small quantities of monoclonal lambda free light chains. In view of the aforementioned results, a bone marrow aspiration was performed. Morphological analysis of the myelogram revealed normally rich smears, with few megakaryocytes devoid of major morphological abnormalities and no excess of blasts. The bone marrow smear showed lymphoid infiltration with 86% pleiomorphic lymphocytes: small mature lymphocytes, nucleolated lymphocytes, and lymphoplasmocytes with basophilic cytoplasm and eccentric nucleus (Figure 3A). Some lymphocytes had small cytoplasmic buds and/or some fine villi, which is characteristic of WM (Figure 3B). Many mast cells (hypogranular and vacuolated) were also observed (Figure 3C).

**Figure 4:**
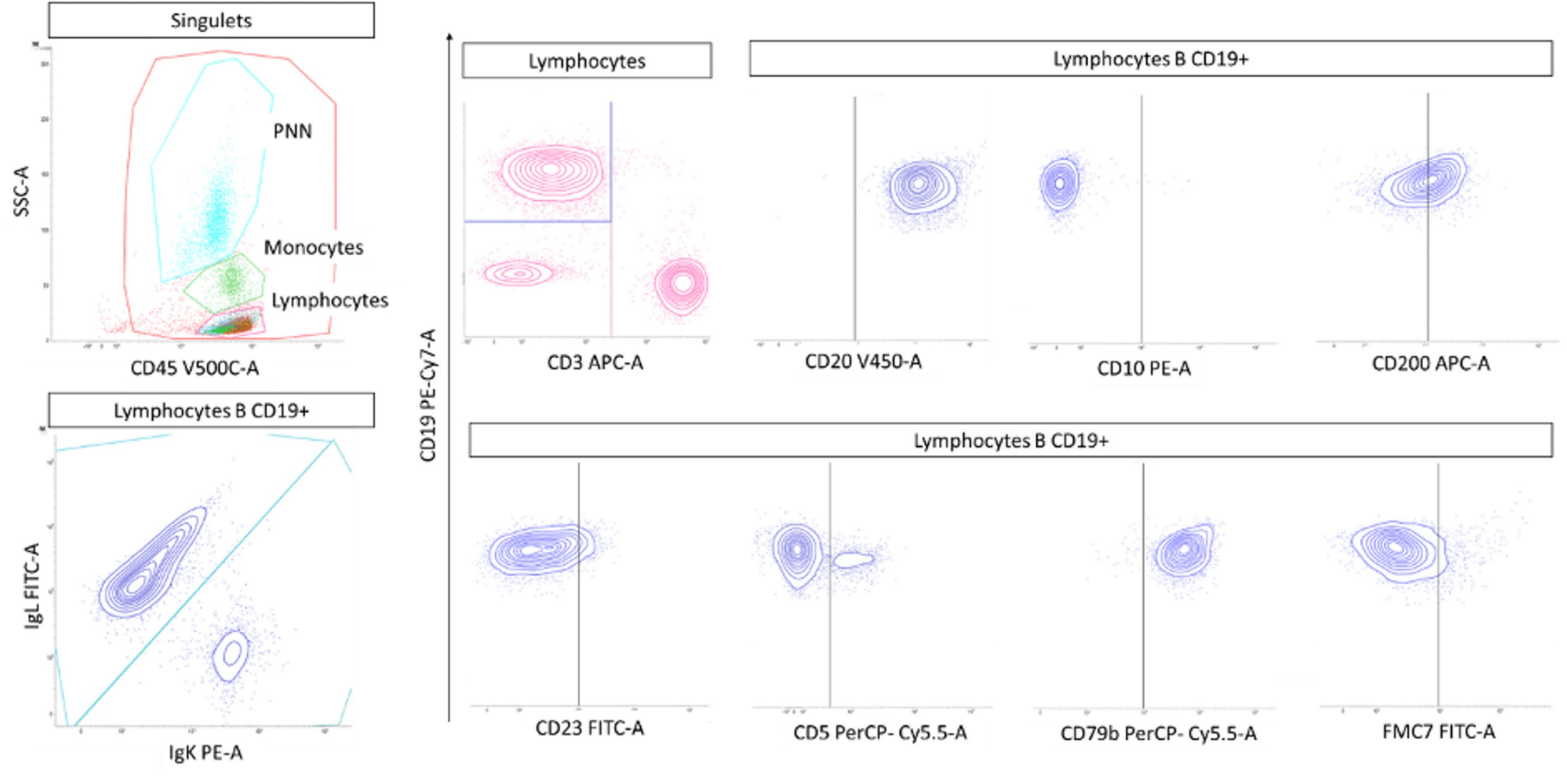
Flow cytometric immunophenotyping of bone marrow: FACSLyric™ biparametric histograms Bone marrow immunophenotyping analysis revealed a monoclonal (lambda) proliferation of B lymphocytes (CD19+, CD20+) with CD5-, CD23+/-, CD79b+, FMC7- and CD10-. FACSLyric™: Becton Dickinson®, NJ, US

Bone marrow immunophenotyping analysis revealed a monoclonal proliferation of CD5-, CD23+/-, and CD10- lambda B lymphocytes (Figure 4). Taken together, these morpho-immunophenotypic features in addition to the presence of monoclonal IgM in the serum denoted WM. Molecular biology analysis revealed a MYD88c.755T>C (p.Leu252Pro) missense mutation and a CXCR4c.1013C>G (p.Ser338Ter) nonsense mutation.

**Figure 5:**
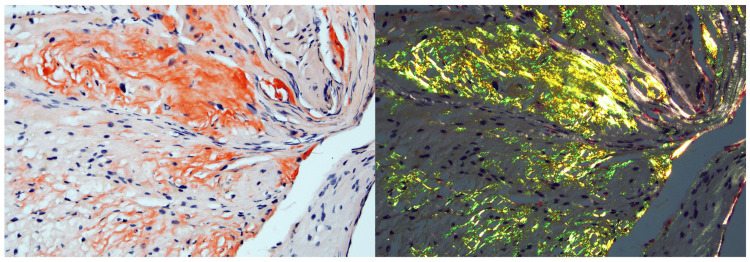
Endomyocardial biopsy Amyloid deposits appeared as a red extracellular substance under light microscopy (Congo red staining x200). Under cross-polarized light, the deposits exhibited a characteristic green birefringence.

Besides, histological examination of the endomyocardial biopsy after Congo red staining with apple-green birefringence under polarized light confirmed the diagnosis of cardiac amyloidosis (Figure 5). Immunohistochemical analysis revealed advanced myocardial lambda Ig light chain deposition, confirming the cardiac AL amyloidosis associated with the WM.

**Figure 6:**
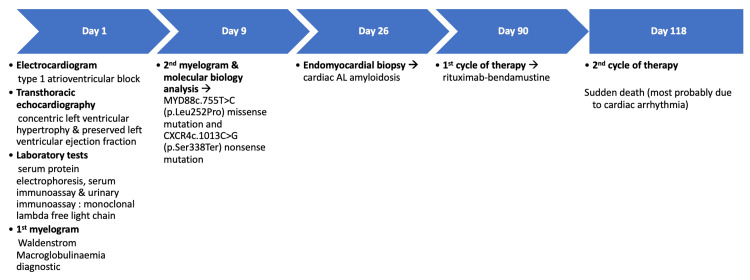
Timeline chart summarizing the key diagnostic and therapeutic steps in this patient's case

Being recommended as first-line therapy in WM, chemotherapy with rituximab and bendamustine was initiated. Yet, unfortunately, the patient died suddenly after the second course of treatment, most probably due to cardiac arrhythmia. No autopsy was performed. Figure 6 chronologically summarizes the key events, diagnostic procedures, and treatment of the patient.​

## Discussion

Amyloidoses belong to a heterogeneous group of diseases linked to the extracellular deposit of insoluble fibrillar proteins in one or more organs. When these proteins are represented by misfolded Ig light chains, it is termed AL amyloidosis. It is secondary to lambda-type Ig light chains in >80% of cases, forming extracellular aggregates organized in folded beta sheets. Proteinaceous deposit in heart tissue, known as cardiac amyloidosis, is part of this systemic disease. AL cardiac amyloidosis has the worst prognosis compared to other amyloid subtypes (e.g., ATTR amyloidosis). Infiltration of all cardiac structures is possible (ventricles, atria, conductive tissue, vessels, valves), resulting in a wide variety of clinical profiles such as heart failure, conductive disorders, or even cardioembolic events [4]. The non-specificity and heterogeneity of clinical presentations complicate the diagnosis. However, it should be suspected when symptoms of heart failure with left ventricular hypertrophy in the absence of aortic stenosis or hypertension are present [5]. Other signs, such as macroglossia and carpal tunnel syndrome (present in our case), may also be observed [6]. Diagnosis is confirmed by an anatomopathological exam, yet the International Amyloidosis Society has fixed two criteria for defining cardiac involvement in AL amyloidosis: myocardial hypertrophy >12 mm and a serum NT-proBNP concentration >332 ng/L [7,8]. It is worth mentioning that the NT-proBNP level is not markedly elevated in our case despite the advanced cardiac amyloidosis. This could be explained by the early-stage kidney disease of the patient (eGFR 78 mL/min/1.73 m^2^) and the known inverse correlation between NT-proBNP and eGFR [9]. Cardiac magnetic resonance imaging findings (i.e., late gadolinium enhancement) and nuclear imaging with bone tracers (to differentiate AL from ATTR amyloidosis) could also be used to refine the diagnosis.

AL amyloidosis is caused by plasma-cell dyscrasias. The monoclonal gammopathy is a plasmacytic proliferation (monoclonal gammopathy of undetermined significance (MGUS), multiple myeloma) in more than 90% of cases and a lymphoplasmacytic proliferation in less than 10% of cases [10]. Thus, in the presence of symptoms suggesting cardiac amyloidosis, it is essential to look for a monoclonal B-cell hemopathy. According to the World Health Organisation (WHO) classification of 2022, WM is a rare low-grade B-cell non-Hodgkin's lymphoma, representing 1% to 2% of hematological malignancies [11]. Diagnosis is based on the presence of bone marrow infiltration by lymphoplasmocytes with specific immunophenotypic and molecular profiles. According to the National Comprehensive Cancer Network (NCCN) Clinical Practice Guidelines in Oncology, WM lymphoplasmocytes are typically negative for CD5 (which helps differentiate it from chronic lymphocytic leukemia), CD10, and CD23, in addition to being CD19+ and CD20+. The positivity of CD23 (10-20% of cases) should not exclude the diagnosis of WM [12]. Mast cells are large, rounded cells with numerous dense granules covering the cytoplasm and nucleus. Although an excess of mast cells is not specific to WM, they are involved in tumor pathophysiology through the expression of the CD40 ligand, a potent inducer of malignant B-cell proliferation [13]. However, their exact role still needs to be elucidated.

No mutation is specific to WM, but the MYD88 and CXCR4 genes are mutated in 95% and 30-40% of the cases, respectively [14]. This was the case with our patient. The MyD88 protein is physiologically present in the cytoplasm of immune system cells. The MYD88 gene encodes two functional protein domains, the C-terminal toll-like/Il-1 receptor (TIR) domain and the N-terminal death domain (DD), involved in myddosome formation. MYD88 is implicated in pro-survival signal transmission and is part of the myddosome, which is formed of 6 MYD88 DDs, 4 IRAK 1 (or IRAK 2, a protein kinase of the family of pto kinases) DDs, and 4 IRAK4 DDs [15]. The myddosome activates the NF-kappaB and AP-1 signaling pathways via MAP kinases. The gain-of-function mutation MYD88L252P induces spontaneous myddosome formation, resulting in constitutive activation of NF-kappaB, thus leading to increased DNA transcription, proliferation, and cell survival. CXCR4, receptor of the chemokine SDF-1 (stromal cell-derived factor 1), is expressed on the surface of numerous cells such as hematopoietic progenitors and lymphocytes. CXCR4/SDF-1 interaction promotes cell migration toward areas where SDF-1 is expressed and plays a role in stem cell homing toward the bone marrow [16]. Around 40 gain-of-function mutations have been identified in a hotspot region encoding the C-terminal domain of CXCR4. These mutations play an important role in WM pathogenesis and disease progression. Indeed, they might impact the clinical presentation and outcome of WM patients, with a more aggressive clinical behavior, and inferior response to Bruton's tyrosine kinase (BTK) inhibitors, namely ibrutinib [17].

Treatment regimens of the association of cardiac AL amyloidosis and WM are derived from those used in WM without concurrent AL amyloidosis or in typical AL amyloidosis with a pure plasma cell neoplastic clone. They are tailored according to the patient's clinical, biological, and mutational profile. Treatment of AL amyloidosis aims at eradicating the underlying cell clone that produces the excess Ig light chains using chemotherapy regimens or autologous stem cell transplantation (in case of young patients who have failed or relapsed after two lines of treatment involving at least the rituximab- and ibrutinib-based regimen) [18]. In parallel, the treatment of WM includes anti-CD20 monoclonal antibody-based combinations and BTK inhibitors. It depends on the need for rapid disease control, the presence of specific disease complications, and the patient's age [19]. Given that CXCR4 mutations may confer resistance to ibrutinib, alternative treatment strategies (other BTK inhibitors, such as zanubrutinib, or proteasome inhibitors) can be considered in such cases [20]. In our patient, a therapy associating rituximab and bendamustine was initiated. Although this was not the case, bortezomib may be associated with reducing the risk of an IgM flare. Unfortunately, therapy efficacy could not be assessed in our patient since he died following the second course of treatment, most probably due to cardiac arrhythmia.

## Conclusions

AL amyloidosis is a multiorgan disease classically described as a ‘great imitator’ because of its heterogeneous, non-specific clinical manifestations. Cardiac tissue involvement, known as cardiac amyloidosis, was, until recently, thought to be incurable and was often only diagnosed at autopsy. While historically challenging to diagnose, advancements in imaging (i.e., cardiac MRI, bone scintigraphy, and endomyocardial biopsy) have significantly improved early detection. The association of cardiac AL amyloidosis with WM remains rare and infrequently reported. The last few years have seen marked progress in elucidating the physiopathology and the diagnosis of such association (mainly via the introduction of next-generation sequencing, improved cardiac imaging techniques, and updated classification criteria). This case report sheds light on this rare combination and underlines the importance of a multidisciplinary collaboration (i.e., between cardiologists, hematologists, clinical pathologists, and amyloidosis specialists) for establishing an early and appropriate diagnosis, thus optimizing patient management.
